# *Notch1* ablation radiosensitizes glioblastoma cells

**DOI:** 10.18632/oncotarget.21409

**Published:** 2017-09-30

**Authors:** Na Han, Guangyuan Hu, Lei Shi, Guoxian Long, Lin Yang, Qingsong Xi, Qiuyun Guo, Jianhua Wang, Zhen Dong, Mengxian Zhang

**Affiliations:** ^1^ Department of Oncology, Tongji Hospital, Tongji Medical College, Huazhong University of Science & Technology, Wuhan, China

**Keywords:** glioblastoma, Notch1, CRISPR/Cas9, radiosensitivity, angiogenesis

## Abstract

Broad specific Notch1 inhibitors suppress glioblastoma multiforme (GBM) growth but have significant gastrointestinal toxicities. Here, we examined Notch1 expression in GBM tissue specimens and its correlation with the overall survival (OS) of GBM patients. Furthermore, using the CRISPR/Cas9 system, we investigated the effects of *Notch1* downregulation on clonogenic growth and angiogenesis of GBM cells and xenografts. Immunohistochemistry showed positive Notch1 expression in 71% (49/69) of GBM tissues. Our multivariate Cox regression analysis further revealed that Notch1 expression was an independent adverse prognostic factor for OS. Notch1 downregulation suppressed the growth of GBM cells U87MG and U251. The mean duration to reach 6 x the starting volume was 18.3 days for xenografts with Notch1 downregulation and 13.4 days for the control xenografts. Immunofluorescent staining further disclosed that Notch1 downregulation markedly increased the number of γH2AX foci and radiosensitized GBM cells. Notch1 downregulation also impaired angiogenesis and attenuated VEGF and hypoxic response to irradiation in xenografts. In conclusion, *Notch1* ablation inhibited GBM cell proliferation and neovascularization and radiosensitized GBM cells and xenografts, suggesting a pivotal role of Notch1 in tumor growth, angiogenesis, and radioresistance in GBM.

## INTRODUCTION

Glioblastoma multiforme (GBM) is an aggressive brain tumor with a dismal outcome despite the current best therapeutic regimen. The median overall survival (OS) is merely 14.6 months with the majority of patients surviving less than two years, even with the current standard of care with maximal surgical debulking followed by adjuvant radiotherapy and oral temozolomide [1, 2]. The infiltrative and invasive growth pattern of the tumor and highly angiogenic characteristics that define GBM result in a high recurrence rate. Intrinsic or acquired chemoresistance and radioresistance remain a significant therapeutic challenge for GBM patients. A recent study by Doan *et al*. revealed that pediatric GBM expressed high levels of acid ceramidase, especially in radioresistant tumors and acid ceramidase could represent a promising target to radiosensitize GBM [3].

Notch signaling is involved in cellular differentiation, survival, and proliferation. Notch signaling is over-activated in human cancers, such as malignant glioma, breast cancer and colorectal carcinoma [4–7]. A suppressive role Notch1 has also been reported in several cancer types such as skin cancer, myeloid leukemia and glioma [8–10]. Notch1 expression has been found to be positively correlated with tumor progression and poor survival of malignant glioma patients [11, 12]. In GBM, Notch signaling is implicated in cellular responses to hypoxia, angiogenesis and tumor growth [13, 14]. Targeting Notch signaling has been shown to reduce GBM xenograft growth and prolong survival of GBM-bearing mice and may also sensitize GBM to irradiation [15, 16]. However, pharmacological Notch inhibitors do not specifically target Notch receptors, and are associated with significant gastrointestinal toxicity [17].

In the current study, we examined the expression of Notch1 in sixty-nine GBM tissue specimens and studied its correlation with the OS of GBM patients. Furthermore, we used the CRISPR (clustered regularly interspaced short palindromic repeat)/Cas9 (CRISPR-associated nuclease 9) system to ablate the *Notch1* gene in human GBM cells and investigated the effects of *Notch1* downregulation on clonogenic growth and angiogenesis of GBM cells and xenografts. The combination of radiotherapy with a radiosensitizer remains a well-established clinical strategy to overcome radioresistance. We further explored whether *Notch1* downregulation could radiosensitize GBM cells and xenografts.

## RESULTS

### Notch1 is an adverse predictor of outcome of GBM patients

**Figure 1:**
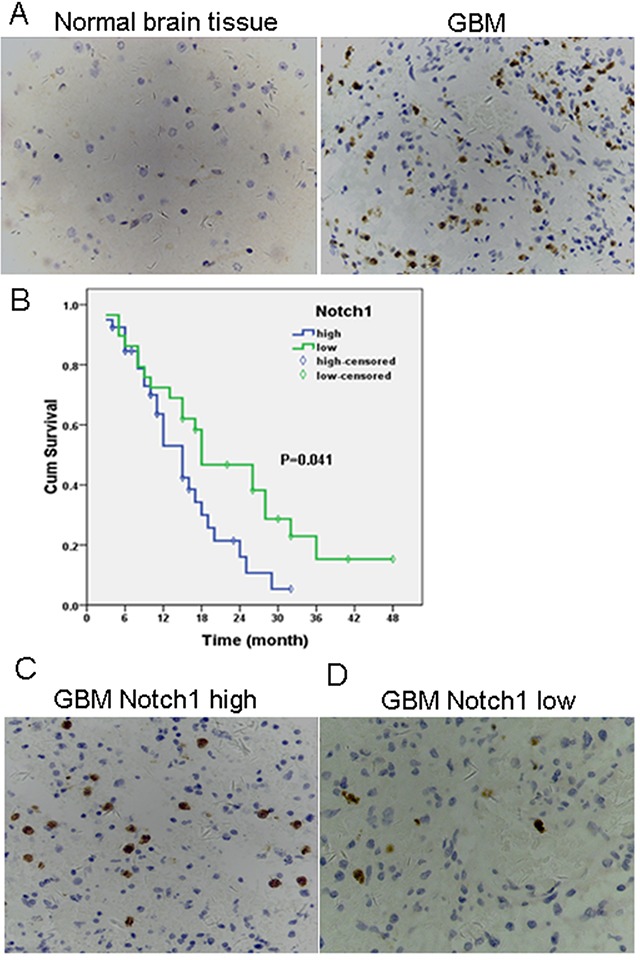
High levels of Notch 1 expression are present in GBM and high tumor Notch 1 expression correlates with worse prognosis **(A)** Immunohistochemical expression of Notch 1 in normal brain tissue (left) and GBM (right). Original magnification 400x; Positive staining = brown; cell nuclei = blue; **(B)** Association between Notch 1 expression and survival time was analyzed using Kaplan-Meier survival analysis in GBM patients when categorized into Notch 1 low and Notch 1 high groups on the basis of staining intensity in IHC. Differences between survival curves were compared using a log-rank test. *P*<0.05 for median survival time. Immunohistochemical expression of Notch 1 in GBM tissues with high **(C)** and low Notch 1 expression **(D)**. Original magnification 400x; Positive staining = brown; cell nuclei = blue.

We examined Notch1 expression in 69 glioma tissue specimens and 8 normal brain tissues pecimens by immunohistochemistry. The demographic and baseline characteristics of the glioma patients are shown in Supplementary Table 1. Notch1 was positively expressed in 12.5% (1/8) of normal brain tissues and 71% (49/69) of GBM tissues (*P*<0.05), as shown in Supplementary Table 2. Furthermore, 58% of the patients had high Notch1 expression. Kaplan-Meier analysis showed a significant negative correlation between Notch1 expression and OS of GBM patients (Figure 1). The median OS of patients with low Notch1 expression (26 months; 95%CI, 17.6–34.5) was significantly longer than that of patients with high Notch1 expression (15 months; 95%CI, 11.8–18.2; *P*=0.002). Multivariate Cox regression analysis revealed that Notch 1 expression was an independent prognostic factor for OS (*P*=0.034) (Supplementary Table 3). These findings indicate that Notch1 was aberrantly expressed in GBM tissues and its expression negatively correlated with OS of GBM patients.

### CRISPR/Cas9 effectively downregulates Notch1 expression

**Figure 2:**
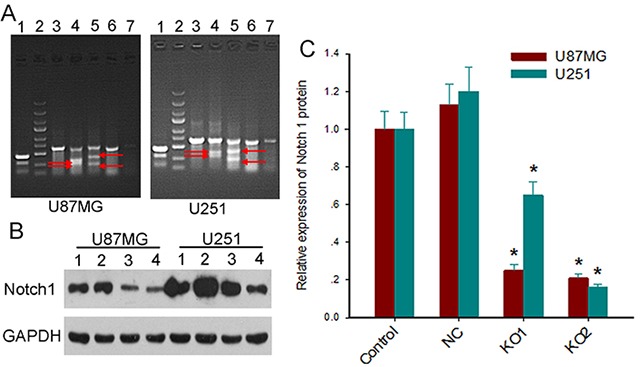
Lentiviral vectors expressing the CAS9 and sgRNAs efficiently knock out *Notch1* gene **(A)** Representative Surveyor assay of genomic DNA isolated from U87MG (left) and U251cells (right) expressing Cas9 and sgRNAs (line 1, positive control; line 2, marker; line 3, NC1; line 4, KO1; line 5, KO2; line 6, NC2; line 7, KO3). The red arrows indicate the cleavage bands. **(B)** The expression levels of Notch1 protein in glioblastoma cells expressing Cas9 and sgRNAs were measured by Western blot (line 1, control; line 2, NC; line 3, KO1; line 4, KO2). **(C)** Quantitative comparison of Notch 1 protein expression measured by Western blot. Notch1 protein expression was calculated by normalizing Notch1 intensity to GAPDH intensity. Data are expressed as mean ± SD; ^*^, *P*<0.05 versus control and NC.

We co-infected GBM cells U87MG and U251 with lentiviruses expressing Cas9 and Notch1-targeting sgRNAKO1, KO2, KO3 or NC. Surveyor assays showed cleavage of the *Notch1* locus at the targeted positions by sgRNA-KO1 and KO2, but not by sgRNA-KO3 or NC (Figure 2A). Immunoblotting assays further revealed significant Notch1 downregulation (>70%) by KO1 or KO2 sgRNAs (Figure 2B and 2C). We used polyclonal U87MG and U251 cells transduced with lentiviruses expressing Cas9 and sgRNA-KO2 for subsequent studies.

### Notch1 downregulation suppresses growth of tumor and radiosensitizes GBM cells

**Figure 3:**
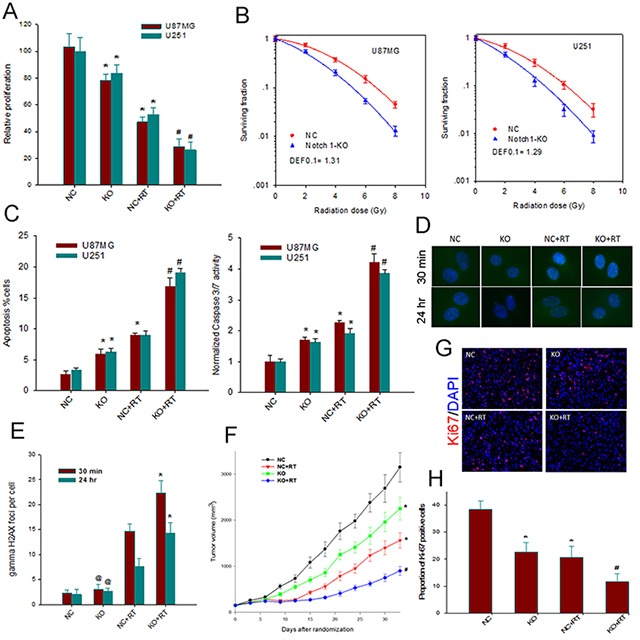
Notch1 downregulation suppresses growth of tumor and radiosensitizes GBM cells **(A)** The effect of Notch 1 knockout on the proliferation of U87MG and U251 cells. Relative numbers of cells are shown as histogram. Columns, mean; bars, SD; RT, radiation therapy ^*^, *P*< 0.05 versus NC; #, *P*< 0.05 versus NC, KO and NC+RT. **(B)** the effect of Notch 1 knockout on the clonogenic survival of U87MG and U251 cells. The survival fraction of both U87MG and U251 cells was significantly decreased in KO group as compared with NC group (p < 0.05). DEF, dose enhancement factor. **(C)** Notch 1 downregulation enhances apoptosis (left) and Caspase 3/7 activity (right) of both irradiated and non-irradiated U87MG and U251 cells. Columns, mean; bars, SD;^*^, *P*<0.05 versus NC; #, *P*< 0.05 versus NC, KO and NC+RT. **(D)** Representative images of γH2AX foci immunofluorescent staining obtained from control and treated cells at 30 min and 24 h after radiation in U87MG. **(E)** The mean number of γH2AX foci at 30 min and 24 h after radiation in U87MG was compared. Columns, mean; bars, SD; ^*^, *P*<0.05 versus NC+RT; @, *P*>0.05 versus NC. **(F)** *In vivo* growth of U87MG tumor xenografts. Data, means ± SE; n=10 animals in each group. ^*^, *P*<0.05 versus NC; #, P < 0.05 versus NC, KO and NC+RT. **(G)** Immunohistochemistry evaluation of the effects of Notch 1 knockout and radiation on the proliferation within U87MG xenografts. Representative examples for the detection of Ki-67 in tumor sections from various treatment groups (200x). **(H)** Quantitative comparison of the Ki-67 index in the tumor sections from various groups of animals.^*^, *P*< 0.05, versus NC; #, *P*< 0.05 versus NC+RT.

We first examined the effect of Notch1 downre-gulation on the proliferation of GBM cells. Trypan blue staining assays showed markedly reduced proliferation of sgRNA-KO2 U87MG and U251 cells compared to the control cells (*P*< 0.05) (Figure 3A). This subdued proliferative response was further observed in sgRNA-KO2 U87MG and U251 cells compared to the control cells when a single dose of 6MV X-ray irradiation of 4 Gy was delivered.

We then determined whether Notch1 downre-gulation affected clonogenicity of GBM cells in response to irradiation. Clonogenic assays demonstrated that CRISPR/Cas9 mediated Notch1 downregulation caused a significant reduction in clonogenic survival of sgRNA-KO2 U87MG and U251 cells compared to the control cells at the indicated irradiation doses, with a dose enhancement factor of 1.31 and 1.29 at a surviving fraction of 10%, respectively (Figure 3B). Next, apoptosis induction was measured by Annexin V-FITC staining and Caspase 3/7 assay. Notch 1 downregulation increased Annexin V-positive cells and caspase 3/7 activity of non-irradiated U87MG and U251 cells (Figure 3C). Moreover, the combination of Notch 1 downregulation and radiation therapy with a dose of 8 Gy resulted in a substantial increase of Annexin V-positive cells and caspase 3/7 activity, in a supra-additive manner. Thus, it appeared that Notch 1 downregulation may radiosensitize GBM cells by augmenting the apoptotic cell death. Enumeration of γH2AX foci by immunofluorescent staining further disclosed that Notch1 downregulation was associated a marked increase in the number of γH2AX foci at 30 min and 24 hours post irradiation (Figures 3D and 3E), suggesting the presence of an increased number of DNA double-strand breaks (DSBs) and impaired DSB repair.

Mouse xenograft assays additionally showed that, consistent with the *in vitro* data, Notch1 downregulation markedly suppressed xenograft growth: the mean duration to reach 6 x the starting volume was 18.3 days for xenografts with Notch1 downregulation and 13.4 days for the control xenografts (*P*<0.05) (Figure 3F). Immunohistochemical staining of mouse xenografts for Ki-67 further showed lower levels of Ki-67 in xenografts with Notch1 downregulation compared to controls regardless of irradiation (Figures 3G and 3H). These findings together demonstrated that Notch1 downregulation could exert growth inhibitory effects on GBM cells and may radiosensitize GBM cells *in vitro* and *in vivo*.

### Notch1 downregulation impairs angiogenesis and attenuates VEGF and hypoxic response to irradiation

**Figure 4:**
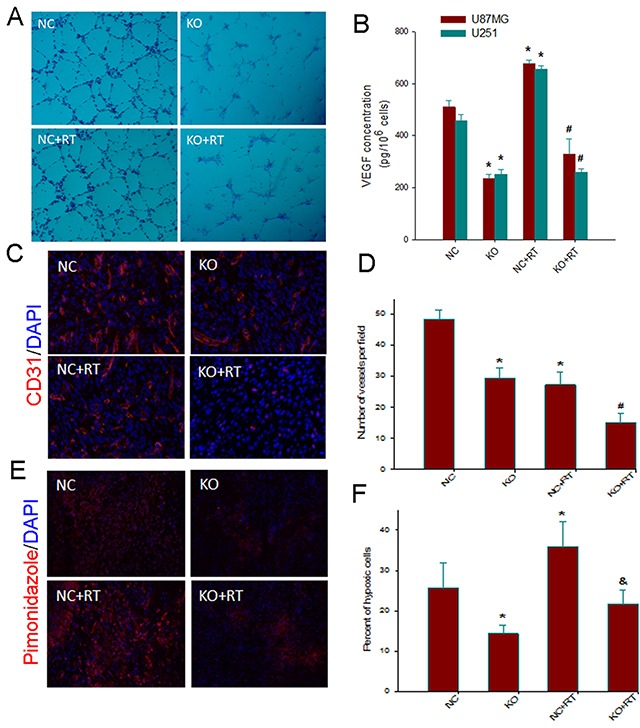
Notch1 downregulation impairs angiogenesis and attenuates VEGF and hypoxic response to irradiation **(A)** Tube-like structure formation ability of human umbilical veins endothelial cells (HUVECs) that were co-cultured with conditioned medium derived from Notch 1-KO or NC U87MGcells, treated with or without 4Gy irradiation, was measured. After incubation, endothelial cells were fixed, and tube-like structures were photographed (100×). **(B)**. Effects of Notch1 knockout on the expression of VEGF in U87MG and U251 cells. Notch 1-KO and NC cells were treated with or without 4Gy irradiation. Sampleswere collected at 24 h post-radiation. VEGF protein levels in the culture supernatant were determined by ELISA. Columns, mean; bars, SD; ^*^, *P*< 0.05, versus NC; #, *P*< 0.05 versus NC+RT. **(C)** Representative examples for the IHC staining of CD31 in tumor sections from various treatment groups (200 x). **(D)** Quantitative comparison of micro-vessel density in the tumor sections from various groups of animals. **(E)** Representative examples for the IHC staining with an antibody for the hypoxic marker pimonidazole in tumor sections from various treatment groups (200 x). **(F)** Quantitative comparison of percent of hypoxic cells in the tumor sections from various groups of animals. Columns, mean; bars, SD; ^*^, *P*< 0.05 versus control; #, *P*< 0.05 versus NC, KO and NC+RT; &, *P*< 0.05 versus NC+RT.

We then sought to elucidate the effect of Notch1disruption on angiogenesis *in vitro*. Tube formation assays showed that compared with that from NC U87MG cells, the culture supernatant of sgRNA-KO2 U87MG cells significantly impaired capillary tube-like structures from HUVECs regardless of irradiation (Figure 4A). ELISA further showed significantly lower production of secreted VEGF in sgRNA-KO2 U87MG cells *versus* the NC cells. Irradiation induced a significant increase in the production of secreted VEGF by the NC cells *versus* non-irradiated NC cells (*P*<0.05) (Figure 4B). On the other hand, sgRNA-KO2 U87MG cells failed to mount a significant increase in the production of secreted VEGF in response to irradiation (*P*>0.05 *versus* non-irradiated sgRNA-KO2 U87MG cells and *P*<0.05 *versus* irradiated NC cells). Furthermore, endothelial cell staining for CD31 showed that Notch1 downregulation was associated with a significant reduction in MVD (*P*<0.05 *versus* controls) (Figure 4C and 4D). Noticeably, Notch1 downregulation was associated with significant decrease in MVD in response to irradiation when compared to the control xenografts. Furthermore, pimonidazole staining revealed a significantly lower hypoxic fraction in Notch1-KO2 xenografts compared to the control xenografts (*P*<0.01) (Figure 4E and 4F). The hypoxic fraction remained significantly depressed in Notch1-KO2 xenografts *versus* the control xenografts in response to irradiation. These findings indicated that Notch1 downregulation compromised angiogenesis and could attenuate VEGF production and hypoxic response upon irradiation.

## DISCUSSION

GBM is an extremely rapidly progressing brain tumor [21] and the majority of GBM patients succumb within two years despite intensive conventional therapeutic protocols. In the current study, we used Notch1 as a novel therapeutic target and found that CRISPR/Cas9 mediated *Notch1* downregulation significantly compromised the growth of GBM cells and xenografts. Furthermore, Notch1 downregulation sensitized GBM cells and xenografts to irradiation. Notch signaling is pivotal to cellular fate determination, differentiation, survival, and proliferation and has been shown to promote glioma growth and enhance radioresistance. Our targeted ablation of Notch1 leading to reduced GBM growth and enhanced radiosensitivity offers further evidence of a role of Notch1 in glioma pathogenesis and in cellular response to irradiation damages.

Dysregulation of the canonical Notch signaling pathway has been documented in a variety of human cancers [5, 22], including malignant gliomas [4, 12, 23, 24]. In the current study, we showed that Notch1 was upregulated in GBMs *versus* normal brain tissues (71% vs. 12.5%, *P*<0.05), and high Notch1 expression correlated with a markedly lower OS of GBM patients. Our results are consistent with the studies by Li *et al* [11]. and Jiang *et al* [25]., indicating that Notch1 is an independent adverse prognostic predictor of the clinical outcome of GBM patients.

Chemoresistance and radioresistance remain important therapeutic challenges in GBM. Current best therapeutic regimen still yields unsatisfactory clinical outcomes, mandating search for novel effective molecular therapeutic targets. Notch signaling has been targeted in many cancer types including GBM [26–28]. However, Notch inhibitors currently in use are broad specific and have unintended side effects such as gastrointestinal toxicities. Our studies and others [15, 16] have shown that inhibition or downregulation of Notch1 sensitized glioma cells to irradiation. The mechanisms whereby Notch affects GBM response to irradiation remain largely unclear. We examined the number of γ-H2AX foci in irradiated GBM cells and found that Notch1 ablation was associated with significant increase in the number of γ-H2AX foci, suggesting the presence of an increased number of DNA DSBs. It remains to be determined whether the increase in the number of DNA DSBs is due to increased formation of DNA DSBs or impaired resolution of DNA DSBs, leading to accumulation of DNA DSBs.

Active angiogenesis is a characteristic feature of GBM, contributing to tumor invasiveness and radioresistance. We found that *Notch1* downregulation caused a significant reduction in MVD in mouse xenografts. Intriguingly, *Notch1* downregulation was associated with a failure to increase in the production of secreted VEGF in response to irradiation. An interaction between Notch and VEGF signaling in tumor angiogenesis has been documented [13, 29]. VEGF is generally considered to be a positive upstream regulator of Notch. Our data suggested the possible presence of a positive feedback-loop regulatory mechanism. Our findings suggest that reduced tumor xenograft growth was at least partially attributed to impaired angiogenesis as a result of *Notch1* downregulation.

Hypoxia is considered to be the driving force of GBM aggressiveness. Hypoxia could increase tumor invasion, resistance to apoptosis, chemoradio-resistance, and tumorigenic cancer stem cell development [30]. Multiple observations showed the connection between hypoxia and Notch signaling [31–33]. Our results showed that inhibiting Notch signaling in GBM xenografts reduced the hypoxic fraction and delayed tumor growth, which further supports crosstalk between Notch signaling and hypoxia and suggests a potential mechanism whereby Notch1 downregulation radiosensitizes GBM cells.

In summary, our findings suggest a pivotal role of Notch1 in tumor growth, angiogenesis, and radioresistance in GBM. CRISPR/Cas9–mediated *Notch1* ablation inhibited GBM cell proliferation and neovascularization and increased radiosensitivity of GBM cells and xenografts.

## MATERIALS AND METHODS

### Cells and tissue acquisition

Human GBM cells U87MG and U251 (ATCC; Manassas, VA) were cultured in DMEM supplemented with 10% fetal calf serum (FCS) and 50 mg/mL penicillin/streptomycin. Primary human umbilical vein endothelial cells (HUVEC, Promocell) were cultured up to passage 10 and maintained in serum reduced (5% FCS) modified Promocell medium supplemented with 2 ng/mL VEGF and 4 ng/mL bFGF.

Fresh GBM tissue specimens were acquired from 69 patients with newly diagnosed GBM who underwent surgery between 1 May, 2012 and 30 December, 2015at Tongji Hospital (Wuhan, China). Eight normal cadaver brain tissue specimens were donated.

### Ethical statement

Acquisition of tissue specimens was approved by the Ethical Committee at Tongji Hospital and was carried out in accordance with state and institutional guidelines on the use of human tissues for experimental purposes and informed consents were signed by all participants.

### Immunohistochemistry

Formalin-fixed, paraffinembedded tissue sections (5μm in thickness) were conventionally prepared from GBM tissue and normal brain tissue specimens. Immunohistochemical staining was performed using polyclonal anti-Notch1 antibody (1:200, Santa Cruz, CA, USA) using the standard avidin-biotin method. The mean percentage of positive cells was scored as follows: 1 for ≤ 25%, 2 for 26-50%, 3 for 51-75%, and 4 for >75%. Negative control was done with substitution of anti-Notch1 antibody with PBS. A positive staining was considered for cells with scores 2 to 4. Staining intensity was scored as follows: negative as 0, weak as 1, moderate as 2, and strong as 3. A final histological score was obtained for each case by multiplying the percentage of positive staining and intensity scores. Glioblastomas with a histological score <5 were considered low expression and those with a histological score ≥ 5 high expression. At least five areas were examined in each tissue section at 400x magnification.

For mouse xenografts, immunohistochemistry was carried out with frozen tumor sections as previously described [18]. After incubation with anti-CD31 (1:100, BD Biosciences PharMingen, CA, USA), anti-Ki-67 (1:200, Abcam, Cambridge, UK) or anti-pimonidazole antibodies (1:100, HPI Inc., Burlington, MA, USA), appropriate fluorescence-labeled secondary antibodies were applied to the slides. Negative control slides were obtained by omitting the primary antibody. Images were captured using a Leica microscope and subsequently analyzed using NIH Image J software. Analysis of tissues was done in at least 5 randomly chosen fields from 3 to 5 sections for each treatment.

### Generation of stable GBM cells by CRISPR/Cas9-expressing lentiviruses targeting Notch1

The third generation lentiviral vector Lenti-Cas9-puro was purchased from Genechem (Shanghai, China). Three sgRNAs (named KO1, KO2, and KO3) targeting *Notch1* were designed using anonline tool (http://crispr.mit.edu) and synthesized (Genechem). The sgRNA oligos were linked into the lentiviralvectorLenti-U6-Puro-GFP and the same vectors with sgRNAs that lack the complete complementary region were used as the negative control (NC). All constructions were verified by Sanger sequencing. Recombinant lentiviral particles were produced by transient transfection of 293T cells and concentrated by ultracentrifugation. U87MG and U251 cells were then infected at a multiplicity of infection of 5followed by puromycin selection for 48 hours. Polyclonal cells stably transfected with *CRISPR*/*Cas9*plasmids were referred as KO 1, 2, or 3 cells, whereas cells stably transfected with the control vector as NC cells.

### Surveyor assays

Surveyor assays were performed as previously described [19]. In brief, cells were incubated at 37°C for 72 hours posttransfection And then genomic DNA was isolated with a DNA extraction kit (Tiangen, Beijing, China) following the manufacturer's protocol. CRISPR targeted site for human *Notch1*was PCR amplified from the purified genomic DNA. PCR products were then denatured by heating to 98°C and slowly re-annealed using a heat block to randomly rehybridize wild type and mutant DNA strands. Samples were then digested at 45°C for 20 minutes with a Knockout and Mutation Detection Kit (GENEsci, Shanghai, China) and resolved by 2% agarose gel electrophoresis. Gel images were obtained with a ChemiDoc XRS system (Bio-Rad, Hercules, CA, USA) and analyzed by Image Lab software (Bio-Rad).

### Trypan blue staining and clonogenic assays

Fifty thousand cells were cultured in a 25 cm^2^ flask overnight and received no irradiation or a single dose of 6MV X-ray irradiation of 4 Gy. After incubation for additional 72 hours, cells were stained with Trypan blue and viable cells were counted. The experiment was performed in triplicate at least three times independently and mean±SME was reported.

The clonogenic assay was carried out as previously described [20]. Increasing numbers of cells (10^2^ to 5 x10^4^) were plated in 25 cm^2^ flasks and irradiated at the indicated doses (0 to 8 Gy) by 6 MV X-ray at a dose rate of 2.5 Gy/min. After 10 to 14 days, colonies were fixed in methanol and stained with 0.5% crystal violet, and the number of colonies containing at least 50 cells was determined and plating efficiency as well as clonogenic survival was calculated. The linear quadratic equation was fitted to data sets to generate survival curves and dose enhancement factor was calculated at 10% surviving fraction.

### Annexin V detection of apoptosis and caspase-3/7 activity assay

An Annexin V-FITC kit (Enzo Life Science, Farmingdale, NY) was used to detect apoptosis according to the manufacturer's instructions. Notch1-KO cells or NC cells were treated with or without 4Gy radiation and were collected 24 hr post-radiation. Cells (10^5^– 10^6^ cells/mL) were washed with cold PBS and resuspended in cold binding buffer containing propidium iodide and Annexin *V-* FITC and incubated on ice for 10 min in the dark, then cells were washed, resuspended and analyzed by flow cytometry (FACScan, Becton Dickinson).

For quantification of Caspase 3/7 activity, a Caspase-Glo®assay (Promega, Mannheim, Germany) was used according to the manufacturer's protocol. Briefly, cells were radiated and seeded in a 96-well plate (2 × 10^4^ cells/well) and incubated for 24 hr. Then, 100 μL of Caspase-Glo 3/7 reagent containing caspase-3/7 substrate was added to each well and the plate was incubated at room temperature for 2 h. Finally, the luminescence of each sample was measured using a GloMaxluminometer (Promega). Results were normalized relative to those of the negative control.

### Western blotting and immunofluorescent staining assays

Cellular extracts were prepared by using RIPA lysis buffer and immunoblotting assays were performed as previously described [20]. Antibodies against Notch1 (1:1000, Rabbit mAb #3439, Cell Signaling Technology) and GAPDH (1:500, sc-32233, Santa Cruz Biotechnology) were used. Specific reactive bands were detected using peroxidase-conjugated secondary antibodies and the immunoreactive bands were visualized by ECL (Amersham, Arlington Heights, IL) and their density was analyzed using NIH ImageJ software (http://rsb.info.nih.gov/ij/).

For immunofluorescent staining, cells were grown and treated in chamber slides at a concentration of 2×10^4^ cells/mL. At specified time points, cells were fixed with 3% paraformaldehyde for 10 min at room temperature, washed three times with PBS, and permeabilized with 0.5% Triton-X-100 for 30 min on ice. Cells were then washed three times with PBS and then three times with 0.5% bovine serum albumin (BSA). AlexaFluor488 anti-H2AX phosphorylated (Ser139) antibody (1:100; #560445, Biolegend, San Diego, CA) was added in 3% BSA and incubated overnight at 4°C. Nuclei were counterstained with 1μg/mL DAPIin PBS for 10 sec. Coverslips were mounted with Fluoromount G solution (SouthernBiotech, Birmingham, AL). Cells were analyzed on a Leica fluorescent microscope. Data were expressed as the mean ± SD of 3 independent experiments in which 50 cells were evaluated.

### Tube formation assay

Twenty-four-well plates were coated with 300 mL Matrigel (BD, Biosciences). HUVECs (5×10^4^) were suspended in 500 μL of conditioned medium from the culture supernatant of Notch1-KO cells or NC cells receiving no or 4Gy irradiation, and then plated onto the polymerized Matrigel and incubated at 37°C for 6 hours. The capillary tube-like structures formed by HUVECs were photographed under a phase contrast inverted microscope.

### ELISA for VEGF

Notch1-KO cells or NC cells were treated with or without 4Gy radiation and then were plated in 6-well tissue culture plates at a density of 1 × 10^6^ cells per well and incubated at 37°C. The supernatants were collected 12h after radiation. VEGF concentration was determined using Quantikine ELISA kits (R&D Systems, MN, USA) according to the manufacturer's instructions.

### Tumor xenograft studies

Human GBM xenografts were established by injecting 5 × 10^6^ Notch1-KO2 U87MG cells or NC cells subcutaneously into the right hind limb of 6 to 8 week-old BALB/c athymic nude mice. After inoculation, the mice were maintained under sterile condition and tumor size was determined once every 3 days by direct measurement with calipers (volume = length x width x width x 0.5). When tumor volume reached approximately 150 mm^3^, animals were randomly assigned to irradiation or sham treatment. Tumors in the irradiation groups were irradiated with a single dose of 8 Gy. Response was evaluated by calculating the time for each tumor to reach 6 times of the baseline size. The hypoxia marker pimonidazole (HPI Inc.) at 60 mg/kg was intraperitoneally injected 1 hour before euthanizing the animals.

Animal studies were approved by the local institutional Animal Care Committee and performed according to the institutional and state guidelines on the care and use of experimental animals.

### Statistical analysis

Fisher's exact test and independent two-tailed *t* test were used for the comparison of parameters between groups according to the variable category. Survival curves were calculated using the Kaplan–Meier method and the comparison of survival curves was performed using the log-rank test. Univariate and multivariate analyses were performed using the Cox regression model to study the effects of Notch 1 expression on OS. A value of *P*<0.05 was considered as statistical significance. Statistical analysis was performed using the software package SPSS 16.0 (SPSS Inc., Chicago, IL, USA).

## SUPPLEMENTARY MATERIALS TABLES



## Abbreviations

GBM: glioblastoma multiforme
OS: overall survival
CRISPR: clustered regularly interspaced short palindromic repeat
Cas9: CRISPR-associated nuclease 9.
