# Supplementary material for: Notch1 ablation radiosensitizes glioblastoma cells
Source: Oncotarget. 2017 Sep 30;8(50):88059–68. doi: 10.18632/oncotarget.21409 (PMC5675693; doi:10.18632/oncotarget.21409)
Supplement: Supplementary file 1 [file oncotarget-08-88059-s001.pdf]

Notch1 ablation radiosensitizes glioblastoma cells

SUPPLEMENTARY MATERIALS

Supplementary Table 1: Characteristics of 69 patients with glioblastoma in association with Notch 1 expression

Characteristics	Notch 1 high	Notch 1 low	*p* value
Age, years			
Medium	53	53	
Range	27-72	38-75	
Age, groups, n (%)			
<40 years	6 (15)	3 (10.3)	0.52
40-60 years	25 (62.5)	16 (55.2)	
≥60 years	9 (22.5)	10 (34.5)	
Gender, n (%)			
Female	14 (35)	10 (34.5)	1.00
Male	26 (65)	19 (65.5)	
Pre-operative KPS, n (%)			
≥70	28 (70)	24 (83)	0.268
<70	12 (30)	5 (17)	
Surgery, n (%)			
Biopsy	4 (10)	1 (3.4)	0.515
Partial resection	15 (37.5)	10 (34.5)	
Subtotal resection	12 (30)	13 (44.8)	
Gross total resection	9 (22.5)	5 (17.3)	
Survival			
Median follow up, months	15	18	
Overall survival, months	15	26	0.002
(95% CI)	11.8-18.2	17.6-34.5	

Supplementary Table 2: Chi-square test of the positive frequency of Notch1 expression between GBM and normal brain tissue

Group	Notch 1 expression		X^2	*p* value
	Positive, n (%)	Negative, n (%)		
GBM	49 (71.0)	20 (29.0)		
Normal brain	1 (12.5)	7 (87.5)	10.780	0.002

Supplementary Table 3: Univariate and multivariate Cox regression analysis of prognostic factors on overall survival in 69 patients with glioblastoma

Vaiables	Uni Cox regression			Multi Cox regression		
	Exp (B) value	95% CI	*p*	Exp (B) value	95% CI	*p*
Age	0.825	0.473-1.437	0.496	1.182	0.655-2.132	0.579
Gender	0.868	0.469-1.606	0.652	0.801	0.425-1.510	0.493
KPS	3.105	1.575-6.124	0.001	3.278	1.507-7.131	0.003
Surgery	0.695	0.471-1.025	0.067	0.607	0.407-0.905	0.014
Notch 1	2.536	1.343-4.787	0.004	2.112	1.059-4.209	0.034
